# Syntheses and crystal structures of the imides 4-(2-phenyl­eth­yl)- and 4-[2-(4-hy­droxy­phen­yl)eth­yl]-4-aza­tetra­cyclo­[5.3.2.0^2,6^.0^8,10^]dodec-11-ene-3,5-dione

**DOI:** 10.1107/S2056989024011253

**Published:** 2024-11-28

**Authors:** Jonathan P. Bajko, Richard J. Staples, Shannon M. Biros

**Affiliations:** a Department of Chemistry Grand Valley State University,Allendale MI 49401 USA; b Center for Crystallographic Research, Department of Chemistry, Michigan State University, East Lansing, MI 48824, USA; Vienna University of Technology, Austria

**Keywords:** crystal structure, imide, tricylic compound, C—H⋯O hydrogen bond, C—H⋯π inter­action

## Abstract

The syntheses and characterization (NMR and XRD) of two substituted [2.2.2]bi­cyclo­octene ring systems are described here. The cyclo­hexene rings of these systems adopt a nearly perfect boat conformation according to analysis using Cremer–Pople parameters. Both structures contain a nearly planar imide ring that is oriented *endo* relative to a bridgehead cyclo­propyl ring. 4-(2-Phenyl­eth­yl)-4-aza­tetra­cyclo­[5.3.2.0^2,6^.0^8,10^]dodec-11-ene-3,5-dione, C_19_H_19_NO_2_, is substituted with a phenylethyl group that hosts C—H⋯π inter­actions in the crystal. 4-[2-(4-Hy­droxy­phen­yl)eth­yl]-4-aza­tetra­cyclo­[5.3.2.0^2,6^.0^8,10^]dodec-11-ene-3,5-dione, C_19_H_19_NO_3_, bears a 4-hy­droxy­phenylethyl group on the imide ring and contains O—H⋯O and C—H⋯O hydrogen bonds.

## Abstract 2

The crystal structures of the title compounds feature C—H⋯π inter­actions as well as O—H⋯O and C—H⋯O hydrogen bonds.

## 1. Chemical context

**Figure 1:**
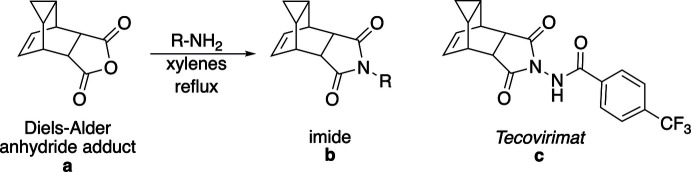
Structures of compounds **a**–**c** related to this work.

Diels–Alder adduct **a** was first reported in 1939 (Fig. 1 ▸; Kohler *et al.*, 1939 ▸), however its structure was not unambiguously determined until nearly 15 years later (Alder & Jacobs, 1953 ▸). In 2020, Winchester and co-workers reported the crystal structure of **a** in this journal (Hulsman *et al.*, 2020 ▸). Anhydride **a** can be easily modified to give the corresponding imides **b** by heating with a primary amine. Previous work has shown that this rigid, tricyclic structure can serve as a scaffold for the design of new compounds with anti­viral activity. One inter­esting imide derivative is compound **c**, Tecovirimat (Bailey *et al.*, 2007 ▸; Hughes 2019 ▸), which has been approved as a treatment for smallpox.

Our inter­est in this chemistry is the use of this series of reactions in an upper-level undergraduate advanced organic chemistry laboratory course (Kurtz & Johnson, 1989 ▸). The tricyclic Diels–Alder adduct **a** is pedagogically useful for in-depth analysis by NMR spectroscopy, and the imides **b** are easily prepared and have turned out to be crystalline. In this context, we report here the syntheses, NMR characterizations and crystal structures of two of these imides, **I**, C_19_H_19_NO_2_, and **II**, C_19_H_19_NO_3_.
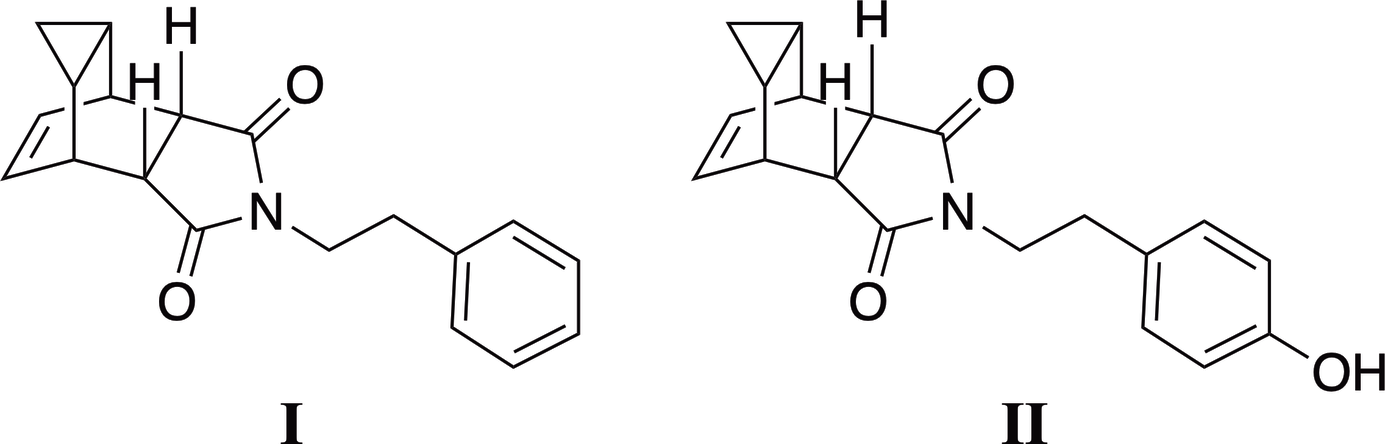


## 2. Structural commentary

**Figure 2:**
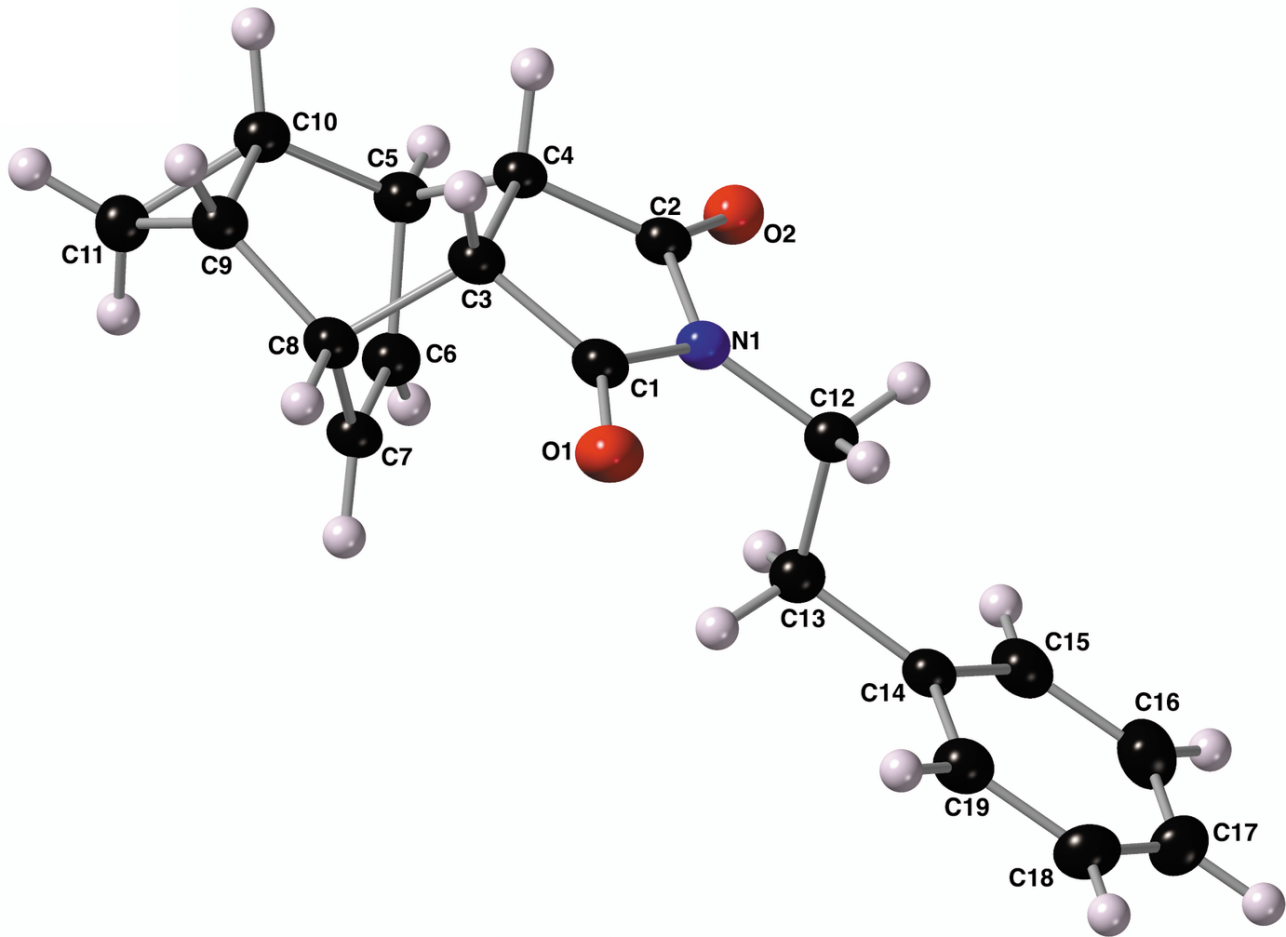
The mol­ecular structure of compound **I** along with the atom-labeling scheme. Displacement ellipsoids are shown at the 50% probability level using standard CPK colors. Hydrogen atoms are shown as spheres of arbitrary size.

The mol­ecular structure of compound **I** is shown in Fig. 2 ▸ along with its atom-labeling scheme. The imide ring (–N1–C1–C3–C4–C2–) is nearly planar with a Cremer–Pople τ value of 1.8 (Cremer & Pople, 1975 ▸) and is oriented *endo* relative to the bridgehead cyclo­propyl ring (–C9—C10—C11–). The carbonyl groups of the imide rings have bond lengths of 1.216 (2) and 1.214 (2) Å, with C—N bond lengths of 1.383 (2) and 1.390 (2) Å in the ring. The phenyl­ethyl substituent on the imide nitro­gen N1 is oriented in a nearly perfect *anti* conformation around the C12—C13 bond with an N1—C12—C13—C14 torsion angle of −177.61 (13)°. The N1—C12 bond is slightly longer than the N—C(O) bond at 1.458 (2) Å. The cyclo­propane ring has C—C bond lengths ranging from 1.507 (3) to 1.515 (2) Å with C—C—C bond angles ranging from 59.71 (12) to 60.29 (12)°. The cyclo­hexene ring system (-C3–C8–C7–C6–C5–C4-) has Cremer–Pople puckering parameters of 90.46 (13) and 299.31 (13)° for θ and φ, respectively, indicating that this ring is in a nearly perfect boat conformation. The alkene group (C6=C7) of this cyclo­hexene ring has a bond length of 1.329 (2) Å.

**Figure 3:**
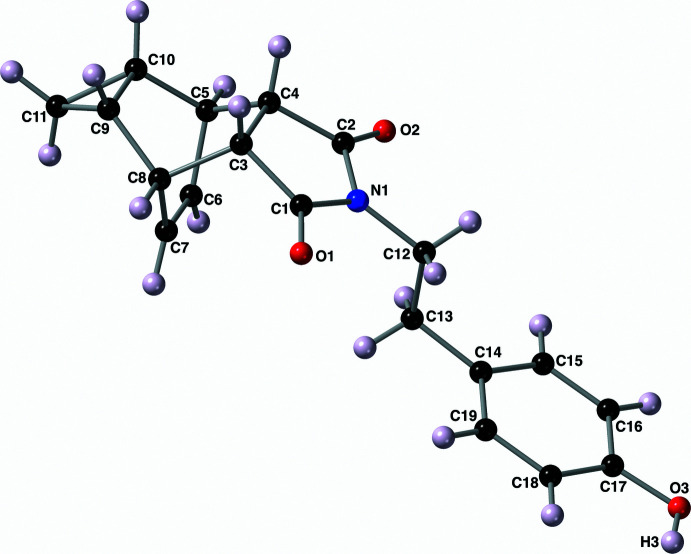
The mol­ecular structure of compound **II** along with the atom-labeling scheme. Displacement ellipsoids are shown at the 50% probability level using standard CPK colors. Hydrogen atoms are shown as spheres of arbitrary size.

The mol­ecular structure of compound **II** (Fig. 3 ▸) is, unsurprisingly, very similar to that of compound **I**. The imide ring (–N1–C1–C3–C4–C2–) again is planar and oriented *endo* relative to the cyclo­propyl bridgehead carbon atoms C9 and C10. The key bond lengths and angles of this compound are nearly identical (within error) to those described above for compound **I**. The 2-ethyl-(4-hy­droxy­phen­yl) substituent bonded to the imide nitro­gen atom N1 again adopts an *anti* conformation around the ethyl C12—C13 bond with an N1—C12—C13—C14 torsion angle of −176.50 (10)°. The hydrogen atom of the phenol group (H3) is nearly coplanar with the atoms of the aromatic ring C14–C19 with a H3—O3—C17–C18 torsion angle of −1.4 (15)°.

## 3. Supra­molecular features

**Figure 4:**
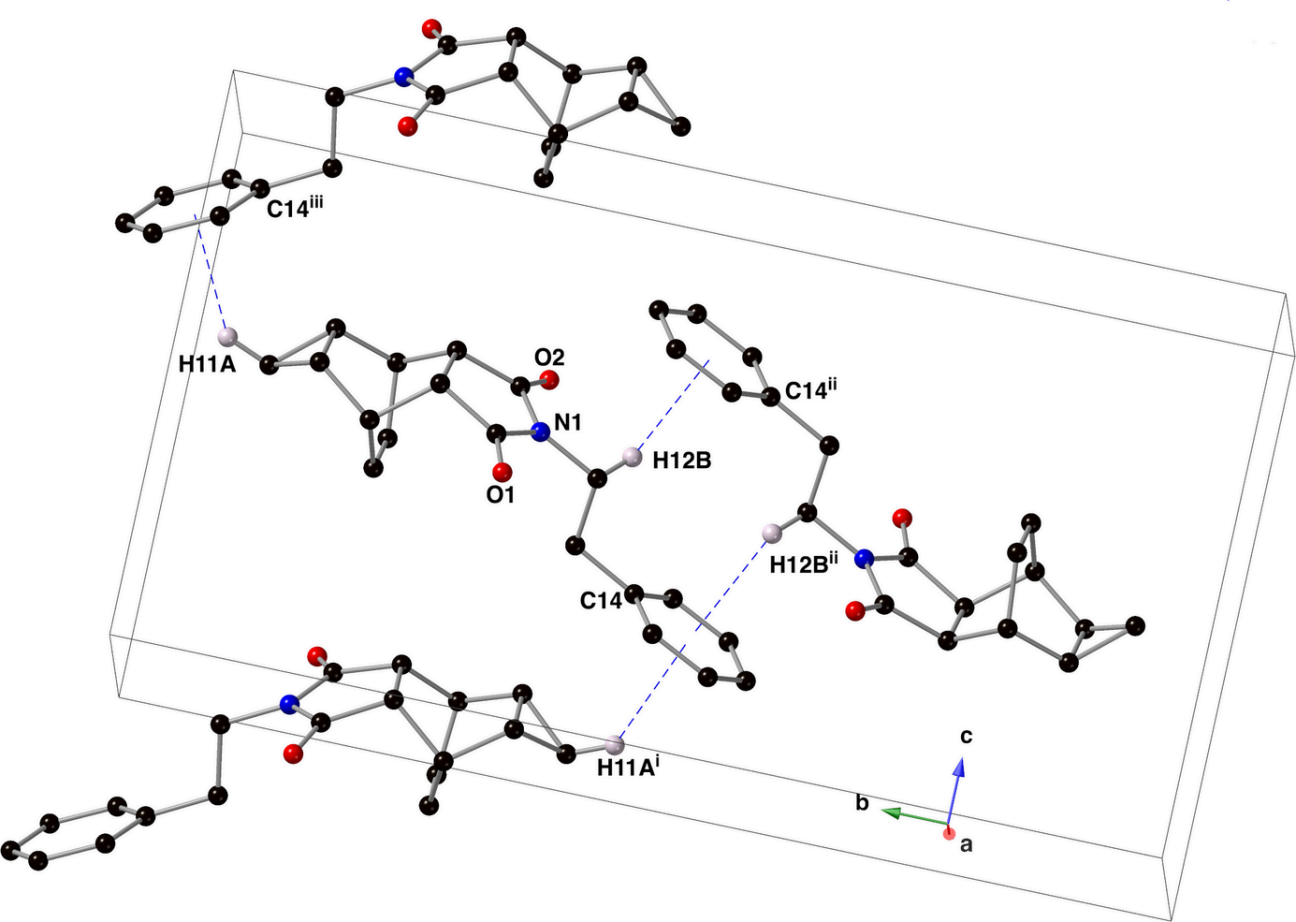
Depiction of C—H⋯π inter­actions (blue dashed lines) present in the crystal of compound **I** using standard CPK colors and a ball-and-stick model. For clarity, only those hydrogen atoms that are involved in a C—H⋯π inter­action are shown. [Symmetry codes: (i) *x* − 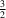, −*y* + 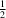, *z* − 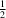; (ii) −*x* + 1, −*y* + 1, −*z* + 1; (iii) *x* − 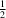, −*y* + 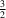, *z* + 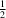.]

The predominant inter­molecular forces present in the crystal of compound **I** are C—H⋯π inter­actions between C11(H11*A*) and C12(H12*B*) and the centroid (*Cg*) of aromatic ring C14–C19 (Fig. 4 ▸, Table 1 ▸). Mol­ecules of compound **I** are arranged into supra­molecular sheets that lie in the *ab* plane.

**Figure 5:**
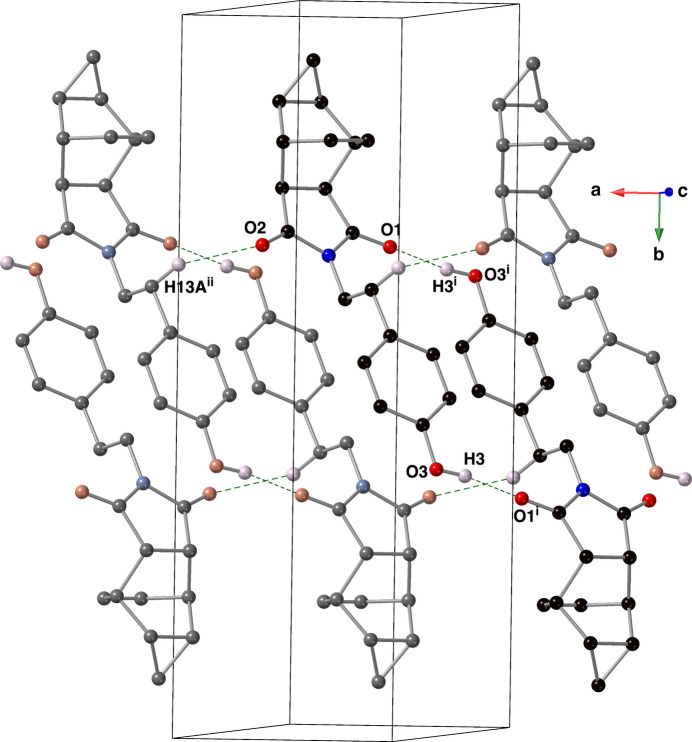
A depiction of the hydrogen-bonded columns present in the crystal of compound **II** using a ball-and-stick model with standard CPK colors. Hydrogen bonds are shown with blue dashed lines; only those hydrogen atoms involved in a hydrogen bond are shown for clarity. [Symmetry codes: (i) −*x*, −*y* + 1, −*z* + 1; (ii) *x -* 1, *y*, *z.*]

The crystal of compound **II** contains classical O—H⋯O and non-classical C—H⋯O hydrogen bonds (Sutor, 1962 ▸, 1963 ▸; Steiner, 1996 ▸) with the carbonyl oxygen atoms O1 and O2 as acceptors (Table 2 ▸). The classical O—H⋯O hydrogen bond has expectedly shorter H—*A* and *D*⋯*A* distances than the C—H⋯O hydrogen bonds. The hydrogen bonds between atoms H3 and O1 form centrosymmetric dimers in the crystal of compound **II**. These dimers are linked into columns *via* the H13*A*⋯O2 inter­actions that run along the *a-*axis direction (Fig. 5 ▸). These columns are then connected into a complex tri-periodic network *via* C—H⋯O inter­actions between H6 and O1.

## 4. Database survey

A search of the Cambridge Structural Database (CSD, version 5.45, updates through June 2024, Groom *et al.*, 2016 ▸) for structures containing the tricyclic ring system shared by compounds **I** and **II** and substituted with carbonyl groups at the appropriate positions returned 16 hits. These hits include anhydride **a** (HOKRIK and HOKRIK01; White & Goh, 2014 ▸; Hulsman *et al.*, 2020 ▸) along with two imides **b** where the substituents are a *p*-bromo­phenyl ring (NUTTEE; Hulsman *et al.*, 2020 ▸) and an isoxazolidine ring (LULVUM; Efremova *et al.*, 2020 ▸). Also found in this subgroup of crystal structures is tecovirimat **c** (UPUDOZ; Zhou *et al.*, 2010 ▸) along with a derivative of **c** where the –CF_3_ group has been replaced with a –Br atom (SOKVIY; Bailey *et al.*, 2007 ▸). An inter­esting hit is VONCEG (Menzek *et al.*, 1991 ▸), which bears a substituted cyclo­hepta­triene ring fused to the tricyclic core of anhydride **a**.

## 5. Synthesis and crystallization

Synthesis of imides **I** and **II**: Diels–Alder anhydride adduct **a** (50 mg, 0.26 mmol) was dissolved in 0.5 ml of xylenes in a small vial at ambient temperature. In a separate small vial at ambient temperature, an equimolar amount of either phenethyl­amine (for **I**) or tyramine (for **II**) was dissolved in 0.5 ml of xylenes and then transferred dropwise to the solution of the anhydride. The reaction mixture was heated to reflux with stirring for 30 minutes and then allowed to cool to room temperature. The stir bar was removed, and the reaction mixture was diluted with slow addition of 5 ml of hexa­nes. The product imide crystallized out of solution upon standing overnight.

**I**: ^1^H-NMR (400 MHz, chloro­form-*d*) δ 7.26 (*m*, 2H), 7.19 (*m*, 3H), 5.59 (*m*, 2H), 3.62 (*m*, 2H), 3.33 (*m*, 2H), 2.90 (*m*, 2H), 2.76 (*t*, *J* = 8.0 Hz, 2H), 1.05 (*m*, 2H), 0.24 (*m*, 1H), 0.19 (*m*, 1H); ^13^C-NMR (100 MHz, chloro­form-*d*) δ 178.5, 137.9, 129.0, 128.5, 127.6, 126.6, 45.3, 39.5, 33.6, 33.5, 9.9, 4.8.

**II**: ^1^H-NMR (400 MHz, DMSO-*d_6_*) δ 9.19 (*s*, 1H, –OH), 6.88 (*d*, *J* = 8.4 Hz, 2H), 6.60 (*d*, *J* = 8.4 Hz, 2H), 5.55 (*m*, 2H), 3.37 (*m*, 2H), 3.13 (*m*, 2H), 2.94 (*m*, 2H), 2.49 (*m*, 2H), 1.06 (*m*, 2H), 0.17 (*m*, 1H), −0.02 (*m*, 1H); ^13^C-NMR (100 MHz, DMSO-*d_6_*) δ 178.5, 156.3, 130.1, 128.5, 127.8, 115.6, 45.1, 40.6, 33.4, 32.6, 10.0, 4.9.

## 6. Refinement

Crystal data, data collection and structure refinement details are summarized in Table 3 ▸. All hydrogen atoms bonded to carbon atoms were placed in calculated positions and refined as riding: C—H = 0.95–1.00 Å with *U*_iso_(H) = 1.2*U*_eq_(C) for methyl­ene, methine, aromatic and alkene groups. In the structure of compound **II**, hydrogen atom H3 (which is part of the hy­droxy group) was located using electron-density difference maps and refined freely.

## Supplementary Material

Crystal structure: contains datablock(s) I, II. DOI: 10.1107/S2056989024011253/wm5740sup1.cif

Structure factors: contains datablock(s) I. DOI: 10.1107/S2056989024011253/wm5740Isup2.hkl

Structure factors: contains datablock(s) II. DOI: 10.1107/S2056989024011253/wm5740IIsup3.hkl

Supporting information file. DOI: 10.1107/S2056989024011253/wm5740Isup4.cml

Supporting information file. DOI: 10.1107/S2056989024011253/wm5740IIsup5.cml

CCDC references: 2403867, 2403866

Additional supporting information:  crystallographic information; 3D view; checkCIF report

## Figures and Tables

**Table 1:** Hydrogen-bond geometry (Å, °) for **I**[Chem scheme1] *Cg* denotes the centroid of the C14-C19 ring.

*D*—H⋯*A*	*D*—H	H⋯*A*	*D*⋯*A*	*D*—H⋯*A*
C11^i^—H11*A*⋯*Cg*	0.99	2.98	3.744 (2)	135
C12^ii^—H12*B*⋯*Cg*	0.99	2.92	3.4608 (19)	115

Symmetry codes: (i) 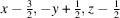; (ii) 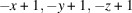.

**Table 2:** Hydrogen-bond geometry (Å, °) for **II**[Chem scheme1]

*D*—H⋯*A*	*D*—H	H⋯*A*	*D*⋯*A*	*D*—H⋯*A*
O3—H3⋯O1^i^	0.92 (2)	1.98 (2)	2.8955 (13)	172 (2)
C13—H13*A*⋯O2^ii^	0.99	2.52	3.4396 (16)	154
C6—H6⋯O1^iii^	0.95	2.50	3.4473 (15)	176

Symmetry codes: (i) 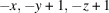; (ii) 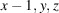; (iii) 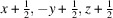.

**Table 3:** Experimental details

	**I**	**II**
Crystal data		
Chemical formula	C_19_H_19_NO_2_	C_19_H_19_NO_3_
*M*_r_	293.35	309.35
Crystal system, space group	Monoclinic, *P*2_1_/*n*	Monoclinic, *P*2_1_/*n*
Temperature (K)	100	100
*a*, *b*, *c* (Å)	6.1340 (2), 21.2794 (8), 11.4373 (3)	6.19482 (10), 20.3854 (3), 12.4574 (2)
β (°)	95.625 (3)	103.3334 (16)
*V* (Å^3^)	1485.70 (8)	1530.76 (4)
*Z*	4	4
Radiation type	Cu *K*α	Cu *K*α
μ (mm^−1^)	0.67	0.73
Crystal size (mm)	0.28 × 0.07 × 0.03	0.25 × 0.05 × 0.03

Data collection		
Diffractometer	XtaLAB Synergy, Dualflex, HyPix	XtaLAB Synergy, Dualflex, HyPix
Absorption correction	Gaussian (*CrysAlis PRO*; Rigaku OD, 2023 ▸)	Gaussian (*CrysAlis PRO*; Rigaku OD, 2023 ▸)
*T*_min_, *T*_max_	0.816, 1.000	0.831, 1.000
No. of measured, independent and observed [*I* > 2σ(*I*)] reflections	11251, 3094, 2481	24225, 3219, 2738
*R*_int_	0.068	0.053
(sin θ/λ)_max_ (Å^−1^)	0.633	0.634

Refinement		
*R*[*F*^2^ > 2σ(*F*^2^)], *wR*(*F*^2^), *S*	0.052, 0.149, 1.05	0.037, 0.099, 1.03
No. of reflections	3094	3219
No. of parameters	199	212
H-atom treatment	H-atom parameters constrained	H atoms treated by a mixture of independent and constrained refinement
Δρ_max_, Δρ_min_ (e Å^−3^)	0.29, −0.32	0.24, −0.19

Computer programs: *CrysAlis PRO* (Rigaku OD, 2023 ▸), *SHELXT* (Sheldrick, 2015*a* ▸), *SHELXL* (Sheldrick, 2015*b* ▸), *CrystalMaker* (Palmer, 2007 ▸) and *OLEX2* (Dolomanov *et al.*, 2009 ▸; Bourhis *et al.*, 2015 ▸).

## supplementary crystallographic information

### 4-(2-Phenylethyl)-4-azatetracyclo[5.3.2.02,6.08,10]dodec-11-ene-3,5-dione (I) Crystal data

**Table:**

C_19_H_19_NO_2_	*F*(000) = 624
*M**_r_* = 293.35	*D*_x_ = 1.311 Mg m^−^^3^
Monoclinic, *P*2_1_/*n*	Cu *K*α radiation, λ = 1.54184 Å
*a* = 6.1340 (2) Å	Cell parameters from 5833 reflections
*b* = 21.2794 (8) Å	θ = 4.1–76.8°
*c* = 11.4373 (3) Å	µ = 0.67 mm^−^^1^
β = 95.625 (3)°	*T* = 100 K
*V* = 1485.70 (8) Å^3^	Irregular, colourless
*Z* = 4	0.28 × 0.07 × 0.03 mm

### 4-(2-Phenylethyl)-4-azatetracyclo[5.3.2.02,6.08,10]dodec-11-ene-3,5-dione (I) Data collection

**Table:**

XtaLAB Synergy, Dualflex, HyPix diffractometer	3094 independent reflections
Radiation source: micro-focus sealed X-ray tube, PhotonJet (Cu) X-ray Source	2481 reflections with *I* > 2σ(*I*)
Mirror monochromator	*R*_int_ = 0.068
Detector resolution: 10.0000 pixels mm^-1^	θ_max_ = 77.2°, θ_min_ = 4.2°
ω scans	*h* = −7→7
Absorption correction: gaussian (CrysAlisPro; Rigaku OD, 2023)	*k* = −26→26
*T*_min_ = 0.816, *T*_max_ = 1.000	*l* = −14→13
11251 measured reflections	

### 4-(2-Phenylethyl)-4-azatetracyclo[5.3.2.02,6.08,10]dodec-11-ene-3,5-dione (I) Refinement

**Table:**

Refinement on *F*^2^	Primary atom site location: dual
Least-squares matrix: full	Hydrogen site location: inferred from neighbouring sites
*R*[*F*^2^ > 2σ(*F*^2^)] = 0.052	H-atom parameters constrained
*wR*(*F*^2^) = 0.149	*w* = 1/[σ^2^(*F*_o_^2^) + (0.0696*P*)^2^ + 0.6093*P*] where *P* = (*F*_o_^2^ + 2*F*_c_^2^)/3
*S* = 1.05	(Δ/σ)_max_ < 0.001
3094 reflections	Δρ_max_ = 0.28 e Å^−^^3^
199 parameters	Δρ_min_ = −0.32 e Å^−^^3^
0 restraints	

### 4-(2-Phenylethyl)-4-azatetracyclo[5.3.2.02,6.08,10]dodec-11-ene-3,5-dione (I) Special details

**Table:**

Geometry. All esds (except the esd in the dihedral angle between two l.s. planes) are estimated using the full covariance matrix. The cell esds are taken into account individually in the estimation of esds in distances, angles and torsion angles; correlations between esds in cell parameters are only used when they are defined by crystal symmetry. An approximate (isotropic) treatment of cell esds is used for estimating esds involving l.s. planes.

### 4-(2-Phenylethyl)-4-azatetracyclo[5.3.2.02,6.08,10]dodec-11-ene-3,5-dione (I) Fractional atomic coordinates and isotropic or equivalent isotropic displacement parameters (Å^2^)

**Table:**

	*x*	*y*	*z*	*U*_iso_*/*U*_eq_	
O1	0.1032 (2)	0.67788 (7)	0.42612 (11)	0.0319 (3)	
O2	0.7405 (2)	0.66698 (6)	0.66530 (11)	0.0330 (3)	
N1	0.4237 (2)	0.65809 (7)	0.54138 (12)	0.0245 (3)	
C1	0.2421 (3)	0.69384 (8)	0.50360 (14)	0.0241 (4)	
C2	0.5649 (3)	0.68831 (8)	0.62579 (15)	0.0256 (4)	
C3	0.2536 (3)	0.75512 (8)	0.56981 (14)	0.0236 (4)	
H3	0.123802	0.759793	0.615613	0.028*	
C4	0.4672 (3)	0.75100 (8)	0.65382 (14)	0.0231 (4)	
H4	0.433452	0.752250	0.737575	0.028*	
C5	0.6221 (3)	0.80617 (8)	0.62777 (15)	0.0246 (4)	
H5	0.763479	0.804214	0.679197	0.030*	
C6	0.6582 (3)	0.80049 (9)	0.49988 (15)	0.0261 (4)	
H6	0.799403	0.795115	0.473882	0.031*	
C7	0.4770 (3)	0.80360 (8)	0.42621 (15)	0.0243 (4)	
H7	0.478006	0.800593	0.343395	0.029*	
C8	0.2696 (3)	0.81229 (8)	0.48505 (15)	0.0236 (4)	
H8	0.138189	0.815029	0.426211	0.028*	
C9	0.2871 (3)	0.86951 (8)	0.56640 (15)	0.0273 (4)	
H9	0.150156	0.882946	0.600205	0.033*	
C10	0.4937 (3)	0.86586 (8)	0.65038 (15)	0.0269 (4)	
H10	0.480218	0.877117	0.734259	0.032*	
C11	0.4636 (3)	0.91838 (9)	0.56136 (16)	0.0306 (4)	
H11A	0.434327	0.961057	0.590545	0.037*	
H11B	0.552811	0.917114	0.493804	0.037*	
C12	0.4735 (3)	0.59815 (8)	0.48821 (15)	0.0266 (4)	
H12A	0.335405	0.575070	0.466226	0.032*	
H12B	0.564550	0.572440	0.546295	0.032*	
C13	0.5946 (3)	0.60743 (9)	0.37955 (16)	0.0281 (4)	
H13A	0.506493	0.634553	0.322642	0.034*	
H13B	0.735919	0.628814	0.401942	0.034*	
C14	0.6365 (3)	0.54518 (8)	0.32266 (14)	0.0243 (4)	
C15	0.8332 (3)	0.51322 (9)	0.34909 (16)	0.0307 (4)	
H15	0.945359	0.531513	0.401466	0.037*	
C16	0.8672 (3)	0.45473 (10)	0.29953 (19)	0.0388 (5)	
H16	1.001862	0.433229	0.318270	0.047*	
C17	0.7048 (4)	0.42793 (10)	0.22293 (19)	0.0410 (5)	
H17	0.727929	0.387999	0.189122	0.049*	
C18	0.5090 (4)	0.45915 (10)	0.19556 (17)	0.0381 (5)	
H18	0.397671	0.440800	0.142784	0.046*	
C19	0.4755 (3)	0.51726 (9)	0.24523 (16)	0.0305 (4)	
H19	0.340323	0.538453	0.226201	0.037*	

### 4-(2-Phenylethyl)-4-azatetracyclo[5.3.2.02,6.08,10]dodec-11-ene-3,5-dione (I) Atomic displacement parameters (Å^2^)

**Table:**

	*U*^11^	*U*^22^	*U*^33^	*U*^12^	*U*^13^	*U*^23^
O1	0.0239 (6)	0.0413 (8)	0.0283 (7)	−0.0053 (5)	−0.0082 (5)	−0.0036 (5)
O2	0.0311 (7)	0.0350 (7)	0.0298 (7)	0.0080 (5)	−0.0131 (5)	−0.0025 (5)
N1	0.0244 (7)	0.0273 (8)	0.0207 (7)	0.0002 (5)	−0.0035 (5)	−0.0007 (5)
C1	0.0200 (7)	0.0305 (9)	0.0212 (8)	−0.0020 (6)	−0.0005 (6)	0.0025 (6)
C2	0.0266 (8)	0.0297 (9)	0.0189 (7)	0.0011 (6)	−0.0061 (6)	−0.0003 (6)
C3	0.0183 (7)	0.0309 (9)	0.0208 (7)	0.0002 (6)	−0.0029 (6)	0.0008 (6)
C4	0.0222 (8)	0.0279 (9)	0.0180 (7)	0.0015 (6)	−0.0043 (6)	0.0000 (6)
C5	0.0193 (7)	0.0299 (9)	0.0227 (8)	−0.0011 (6)	−0.0078 (6)	−0.0010 (6)
C6	0.0201 (8)	0.0313 (9)	0.0266 (8)	−0.0003 (6)	0.0002 (6)	−0.0007 (7)
C7	0.0238 (8)	0.0294 (9)	0.0194 (7)	−0.0011 (6)	−0.0002 (6)	0.0010 (6)
C8	0.0177 (7)	0.0295 (9)	0.0223 (8)	0.0012 (6)	−0.0043 (6)	0.0018 (6)
C9	0.0255 (8)	0.0305 (9)	0.0251 (8)	0.0042 (7)	−0.0021 (7)	0.0009 (7)
C10	0.0276 (8)	0.0291 (9)	0.0226 (8)	0.0004 (7)	−0.0046 (7)	−0.0021 (6)
C11	0.0347 (9)	0.0272 (9)	0.0284 (9)	0.0006 (7)	−0.0040 (7)	0.0001 (7)
C12	0.0294 (8)	0.0267 (8)	0.0226 (8)	−0.0009 (7)	−0.0029 (7)	−0.0011 (6)
C13	0.0298 (8)	0.0282 (9)	0.0261 (8)	−0.0021 (7)	0.0008 (7)	−0.0004 (7)
C14	0.0243 (8)	0.0278 (9)	0.0206 (7)	−0.0008 (6)	0.0005 (6)	0.0021 (6)
C15	0.0242 (8)	0.0378 (10)	0.0296 (9)	0.0004 (7)	−0.0009 (7)	0.0070 (7)
C16	0.0360 (10)	0.0377 (11)	0.0448 (11)	0.0111 (8)	0.0145 (8)	0.0110 (9)
C17	0.0576 (13)	0.0301 (10)	0.0381 (10)	−0.0008 (9)	0.0196 (9)	−0.0033 (8)
C18	0.0474 (11)	0.0378 (11)	0.0284 (9)	−0.0086 (9)	−0.0001 (8)	−0.0063 (8)
C19	0.0286 (9)	0.0348 (10)	0.0266 (8)	−0.0008 (7)	−0.0043 (7)	0.0007 (7)

### 4-(2-Phenylethyl)-4-azatetracyclo[5.3.2.02,6.08,10]dodec-11-ene-3,5-dione (I) Geometric parameters (Å, º)

**Table:**

O1—C1	1.216 (2)	C9—C11	1.507 (3)
O2—C2	1.214 (2)	C10—H10	1.0000
N1—C1	1.383 (2)	C10—C11	1.511 (2)
N1—C2	1.390 (2)	C11—H11A	0.9900
N1—C12	1.458 (2)	C11—H11B	0.9900
C1—C3	1.506 (2)	C12—H12A	0.9900
C2—C4	1.510 (2)	C12—H12B	0.9900
C3—H3	1.0000	C12—C13	1.522 (3)
C3—C4	1.550 (2)	C13—H13A	0.9900
C3—C8	1.565 (2)	C13—H13B	0.9900
C4—H4	1.0000	C13—C14	1.509 (3)
C4—C5	1.557 (2)	C14—C15	1.392 (2)
C5—H5	1.0000	C14—C19	1.393 (2)
C5—C6	1.506 (2)	C15—H15	0.9500
C5—C10	1.530 (2)	C15—C16	1.392 (3)
C6—H6	0.9500	C16—H16	0.9500
C6—C7	1.329 (2)	C16—C17	1.384 (3)
C7—H7	0.9500	C17—H17	0.9500
C7—C8	1.507 (2)	C17—C18	1.381 (3)
C8—H8	1.0000	C18—H18	0.9500
C8—C9	1.530 (2)	C18—C19	1.385 (3)
C9—H9	1.0000	C19—H19	0.9500
C9—C10	1.515 (2)		
			
C1—N1—C2	113.00 (15)	C11—C9—C10	60.00 (11)
C1—N1—C12	123.10 (14)	C5—C10—H10	117.0
C2—N1—C12	123.54 (14)	C9—C10—C5	110.36 (14)
O1—C1—N1	124.01 (17)	C9—C10—H10	117.0
O1—C1—C3	127.11 (16)	C11—C10—C5	122.08 (15)
N1—C1—C3	108.82 (13)	C11—C10—C9	59.71 (11)
O2—C2—N1	123.73 (16)	C11—C10—H10	117.0
O2—C2—C4	127.40 (15)	C9—C11—C10	60.29 (12)
N1—C2—C4	108.83 (14)	C9—C11—H11A	117.7
C1—C3—H3	110.4	C9—C11—H11B	117.7
C1—C3—C4	104.95 (13)	C10—C11—H11A	117.7
C1—C3—C8	111.37 (14)	C10—C11—H11B	117.7
C4—C3—H3	110.4	H11A—C11—H11B	114.9
C4—C3—C8	109.12 (13)	N1—C12—H12A	109.3
C8—C3—H3	110.4	N1—C12—H12B	109.3
C2—C4—C3	104.33 (13)	N1—C12—C13	111.47 (15)
C2—C4—H4	110.5	H12A—C12—H12B	108.0
C2—C4—C5	111.29 (14)	C13—C12—H12A	109.3
C3—C4—H4	110.5	C13—C12—H12B	109.3
C3—C4—C5	109.47 (13)	C12—C13—H13A	109.5
C5—C4—H4	110.5	C12—C13—H13B	109.5
C4—C5—H5	111.5	H13A—C13—H13B	108.1
C6—C5—C4	106.02 (13)	C14—C13—C12	110.83 (15)
C6—C5—H5	111.5	C14—C13—H13A	109.5
C6—C5—C10	110.98 (14)	C14—C13—H13B	109.5
C10—C5—C4	105.06 (14)	C15—C14—C13	121.05 (16)
C10—C5—H5	111.5	C15—C14—C19	118.36 (17)
C5—C6—H6	122.6	C19—C14—C13	120.55 (16)
C7—C6—C5	114.74 (15)	C14—C15—H15	119.7
C7—C6—H6	122.6	C16—C15—C14	120.64 (17)
C6—C7—H7	122.8	C16—C15—H15	119.7
C6—C7—C8	114.39 (15)	C15—C16—H16	120.0
C8—C7—H7	122.8	C17—C16—C15	119.94 (18)
C3—C8—H8	111.5	C17—C16—H16	120.0
C7—C8—C3	106.75 (13)	C16—C17—H17	120.0
C7—C8—H8	111.5	C18—C17—C16	120.10 (19)
C7—C8—C9	110.95 (14)	C18—C17—H17	120.0
C9—C8—C3	104.29 (14)	C17—C18—H18	120.1
C9—C8—H8	111.5	C17—C18—C19	119.83 (18)
C8—C9—H9	116.8	C19—C18—H18	120.1
C10—C9—C8	110.42 (14)	C14—C19—H19	119.4
C10—C9—H9	116.8	C18—C19—C14	121.12 (17)
C11—C9—C8	122.26 (16)	C18—C19—H19	119.4
C11—C9—H9	116.8		
			
O1—C1—C3—C4	−177.86 (17)	C5—C6—C7—C8	0.1 (2)
O1—C1—C3—C8	−59.9 (2)	C5—C10—C11—C9	96.28 (17)
O2—C2—C4—C3	174.98 (18)	C6—C5—C10—C9	51.69 (19)
O2—C2—C4—C5	57.0 (2)	C6—C5—C10—C11	−14.6 (2)
N1—C1—C3—C4	−0.50 (18)	C6—C7—C8—C3	−58.86 (19)
N1—C1—C3—C8	117.44 (15)	C6—C7—C8—C9	54.2 (2)
N1—C2—C4—C3	−2.65 (18)	C7—C8—C9—C10	−52.07 (19)
N1—C2—C4—C5	−120.61 (15)	C7—C8—C9—C11	14.6 (2)
N1—C12—C13—C14	−177.61 (13)	C8—C3—C4—C2	−117.59 (15)
C1—N1—C2—O2	−175.20 (17)	C8—C3—C4—C5	1.61 (18)
C1—N1—C2—C4	2.5 (2)	C8—C9—C10—C5	0.2 (2)
C1—N1—C12—C13	83.9 (2)	C8—C9—C10—C11	116.27 (17)
C1—C3—C4—C2	1.86 (17)	C8—C9—C11—C10	−96.44 (17)
C1—C3—C4—C5	121.06 (15)	C10—C5—C6—C7	−54.2 (2)
C1—C3—C8—C7	−60.75 (16)	C11—C9—C10—C5	−116.06 (17)
C1—C3—C8—C9	−178.27 (13)	C12—N1—C1—O1	2.8 (3)
C2—N1—C1—O1	176.20 (17)	C12—N1—C1—C3	−174.62 (15)
C2—N1—C1—C3	−1.3 (2)	C12—N1—C2—O2	−1.9 (3)
C2—N1—C12—C13	−88.81 (19)	C12—N1—C2—C4	175.86 (15)
C2—C4—C5—C6	57.84 (17)	C12—C13—C14—C15	−94.16 (19)
C2—C4—C5—C10	175.42 (13)	C12—C13—C14—C19	83.6 (2)
C3—C4—C5—C6	−56.97 (17)	C13—C14—C15—C16	177.59 (17)
C3—C4—C5—C10	60.61 (16)	C13—C14—C19—C18	−177.77 (18)
C3—C8—C9—C10	62.51 (17)	C14—C15—C16—C17	0.2 (3)
C3—C8—C9—C11	129.18 (16)	C15—C14—C19—C18	0.1 (3)
C4—C3—C8—C7	54.64 (17)	C15—C16—C17—C18	0.0 (3)
C4—C3—C8—C9	−62.88 (16)	C16—C17—C18—C19	−0.2 (3)
C4—C5—C6—C7	59.37 (19)	C17—C18—C19—C14	0.1 (3)
C4—C5—C10—C9	−62.47 (17)	C19—C14—C15—C16	−0.2 (3)
C4—C5—C10—C11	−128.74 (16)		

### 4-(2-Phenylethyl)-4-azatetracyclo[5.3.2.02,6.08,10]dodec-11-ene-3,5-dione (I) Hydrogen-bond geometry (Å, º)

*Cg* denotes the centroid of the C14-C19 ring.

**Table:**

*D*—H···*A*	*D*—H	H···*A*	*D*···*A*	*D*—H···*A*
C11^i^—H11*A*···*Cg*	0.99	2.98	3.744 (2)	135
C12^ii^—H12*B*···*Cg*	0.99	2.92	3.4608 (19)	115

Symmetry codes: (i) *x*−3/2, −*y*+1/2, *z*−1/2; (ii) −*x*+1, −*y*+1, −*z*+1.

### 4-[2-(4-Hydroxyphenyl)ethyl]-4-azatetracyclo[5.3.2.02,6.08,10]dodec-11-ene-3,5-dione (II) Crystal data

**Table:**

C_19_H_19_NO_3_	*F*(000) = 656
*M**_r_* = 309.35	*D*_x_ = 1.342 Mg m^−^^3^
Monoclinic, *P*2_1_/*n*	Cu *K*α radiation, λ = 1.54184 Å
*a* = 6.19482 (10) Å	Cell parameters from 11000 reflections
*b* = 20.3854 (3) Å	θ = 4.2–77.3°
*c* = 12.4574 (2) Å	µ = 0.73 mm^−^^1^
β = 103.3334 (16)°	*T* = 100 K
*V* = 1530.76 (4) Å^3^	Needle, colourless
*Z* = 4	0.25 × 0.05 × 0.03 mm

### 4-[2-(4-Hydroxyphenyl)ethyl]-4-azatetracyclo[5.3.2.02,6.08,10]dodec-11-ene-3,5-dione (II) Data collection

**Table:**

XtaLAB Synergy, Dualflex, HyPix diffractometer	3219 independent reflections
Radiation source: micro-focus sealed X-ray tube, PhotonJet (Cu) X-ray Source	2738 reflections with *I* > 2σ(*I*)
Mirror monochromator	*R*_int_ = 0.053
Detector resolution: 10.0000 pixels mm^-1^	θ_max_ = 77.7°, θ_min_ = 4.2°
ω scans	*h* = −7→7
Absorption correction: gaussian (CrysAlisPro; Rigaku OD, 2023)	*k* = −25→22
*T*_min_ = 0.831, *T*_max_ = 1.000	*l* = −15→15
24225 measured reflections	

### 4-[2-(4-Hydroxyphenyl)ethyl]-4-azatetracyclo[5.3.2.02,6.08,10]dodec-11-ene-3,5-dione (II) Refinement

**Table:**

Refinement on *F*^2^	Primary atom site location: dual
Least-squares matrix: full	Hydrogen site location: mixed
*R*[*F*^2^ > 2σ(*F*^2^)] = 0.037	H atoms treated by a mixture of independent and constrained refinement
*wR*(*F*^2^) = 0.099	*w* = 1/[σ^2^(*F*_o_^2^) + (0.0479*P*)^2^ + 0.4204*P*] where *P* = (*F*_o_^2^ + 2*F*_c_^2^)/3
*S* = 1.03	(Δ/σ)_max_ = 0.001
3219 reflections	Δρ_max_ = 0.24 e Å^−^^3^
212 parameters	Δρ_min_ = −0.19 e Å^−^^3^
0 restraints	

### 4-[2-(4-Hydroxyphenyl)ethyl]-4-azatetracyclo[5.3.2.02,6.08,10]dodec-11-ene-3,5-dione (II) Special details

**Table:**

Geometry. All esds (except the esd in the dihedral angle between two l.s. planes) are estimated using the full covariance matrix. The cell esds are taken into account individually in the estimation of esds in distances, angles and torsion angles; correlations between esds in cell parameters are only used when they are defined by crystal symmetry. An approximate (isotropic) treatment of cell esds is used for estimating esds involving l.s. planes.

### 4-[2-(4-Hydroxyphenyl)ethyl]-4-azatetracyclo[5.3.2.02,6.08,10]dodec-11-ene-3,5-dione (II) Fractional atomic coordinates and isotropic or equivalent isotropic displacement parameters (Å^2^)

**Table:**

	*x*	*y*	*z*	*U*_iso_*/*U*_eq_	
O1	0.22461 (14)	0.32415 (4)	0.34473 (7)	0.0282 (2)	
O2	0.92166 (14)	0.32894 (5)	0.56437 (8)	0.0312 (2)	
O3	0.22408 (16)	0.63973 (4)	0.76502 (7)	0.0283 (2)	
N1	0.56517 (16)	0.33992 (5)	0.46177 (8)	0.0233 (2)	
C1	0.4153 (2)	0.30627 (6)	0.38185 (9)	0.0226 (2)	
C2	0.7694 (2)	0.30848 (6)	0.49296 (10)	0.0235 (3)	
C3	0.52553 (19)	0.24518 (6)	0.35103 (9)	0.0220 (2)	
H3A	0.532143	0.246899	0.271628	0.026*	
C4	0.76183 (19)	0.24663 (6)	0.42573 (10)	0.0218 (2)	
H4	0.874553	0.248648	0.379854	0.026*	
C5	0.79950 (19)	0.18446 (6)	0.50036 (10)	0.0226 (3)	
H5	0.948741	0.184960	0.552529	0.027*	
C6	0.6156 (2)	0.18277 (6)	0.56081 (10)	0.0238 (3)	
H6	0.642429	0.183069	0.639031	0.029*	
C7	0.4127 (2)	0.18083 (6)	0.49618 (10)	0.0253 (3)	
H7	0.282883	0.179131	0.524435	0.030*	
C8	0.4050 (2)	0.18152 (6)	0.37461 (10)	0.0244 (3)	
H8	0.249038	0.179731	0.329527	0.029*	
C9	0.5462 (2)	0.12572 (6)	0.34451 (10)	0.0261 (3)	
H9	0.534891	0.117643	0.264226	0.031*	
C10	0.7766 (2)	0.12710 (6)	0.41887 (10)	0.0252 (3)	
H10	0.903112	0.119695	0.382893	0.030*	
C11	0.6262 (2)	0.06955 (6)	0.42227 (11)	0.0303 (3)	
H11A	0.562029	0.064960	0.487680	0.036*	
H11B	0.661504	0.027621	0.390032	0.036*	
C12	0.5151 (2)	0.40098 (6)	0.51242 (10)	0.0246 (3)	
H12A	0.390600	0.423331	0.461438	0.030*	
H12B	0.646043	0.430232	0.523776	0.030*	
C13	0.4537 (2)	0.38991 (6)	0.62283 (10)	0.0246 (3)	
H13A	0.327791	0.358846	0.612991	0.030*	
H13B	0.581309	0.370480	0.676067	0.030*	
C14	0.39024 (19)	0.45416 (6)	0.66741 (9)	0.0218 (2)	
C15	0.5441 (2)	0.49292 (6)	0.73957 (10)	0.0252 (3)	
H15	0.690945	0.477136	0.766275	0.030*	
C16	0.4865 (2)	0.55417 (6)	0.77310 (10)	0.0257 (3)	
H16	0.593183	0.579772	0.822688	0.031*	
C17	0.2723 (2)	0.57793 (6)	0.73392 (9)	0.0231 (3)	
C18	0.11533 (19)	0.53920 (6)	0.66448 (10)	0.0242 (3)	
H18	−0.032540	0.554538	0.639392	0.029*	
C19	0.17502 (19)	0.47814 (6)	0.63190 (10)	0.0235 (3)	
H19	0.066654	0.452024	0.584257	0.028*	
H3	0.077 (4)	0.6475 (12)	0.7333 (18)	0.068 (6)*	

### 4-[2-(4-Hydroxyphenyl)ethyl]-4-azatetracyclo[5.3.2.02,6.08,10]dodec-11-ene-3,5-dione (II) Atomic displacement parameters (Å^2^)

**Table:**

	*U*^11^	*U*^22^	*U*^33^	*U*^12^	*U*^13^	*U*^23^
O1	0.0251 (4)	0.0331 (5)	0.0243 (4)	0.0062 (4)	0.0013 (3)	0.0008 (3)
O2	0.0250 (5)	0.0298 (5)	0.0357 (5)	−0.0029 (4)	0.0006 (4)	−0.0067 (4)
O3	0.0302 (5)	0.0249 (5)	0.0298 (5)	0.0027 (4)	0.0071 (4)	−0.0037 (3)
N1	0.0247 (5)	0.0229 (5)	0.0217 (5)	0.0018 (4)	0.0041 (4)	−0.0009 (4)
C1	0.0242 (6)	0.0260 (6)	0.0175 (5)	0.0013 (5)	0.0045 (4)	0.0021 (4)
C2	0.0223 (6)	0.0239 (6)	0.0244 (6)	−0.0012 (4)	0.0058 (4)	0.0003 (5)
C3	0.0224 (6)	0.0249 (6)	0.0183 (5)	0.0011 (4)	0.0036 (4)	−0.0015 (4)
C4	0.0198 (5)	0.0243 (6)	0.0221 (6)	−0.0004 (4)	0.0062 (4)	−0.0008 (4)
C5	0.0196 (5)	0.0247 (6)	0.0229 (6)	0.0009 (4)	0.0037 (4)	−0.0001 (4)
C6	0.0255 (6)	0.0250 (6)	0.0213 (6)	0.0004 (5)	0.0062 (5)	0.0010 (4)
C7	0.0226 (6)	0.0268 (6)	0.0280 (6)	−0.0005 (5)	0.0091 (5)	−0.0004 (5)
C8	0.0202 (6)	0.0271 (6)	0.0244 (6)	−0.0016 (5)	0.0020 (4)	−0.0030 (5)
C9	0.0266 (6)	0.0258 (6)	0.0248 (6)	−0.0012 (5)	0.0039 (5)	−0.0044 (5)
C10	0.0242 (6)	0.0242 (6)	0.0276 (6)	0.0010 (5)	0.0068 (5)	−0.0013 (5)
C11	0.0333 (7)	0.0236 (6)	0.0330 (7)	−0.0012 (5)	0.0059 (5)	−0.0020 (5)
C12	0.0294 (6)	0.0207 (6)	0.0237 (6)	0.0040 (5)	0.0060 (5)	−0.0003 (4)
C13	0.0272 (6)	0.0226 (6)	0.0237 (6)	0.0018 (5)	0.0054 (5)	0.0016 (5)
C14	0.0242 (6)	0.0224 (6)	0.0194 (5)	0.0009 (4)	0.0059 (4)	0.0023 (4)
C15	0.0215 (6)	0.0279 (6)	0.0248 (6)	0.0013 (5)	0.0027 (4)	0.0007 (5)
C16	0.0241 (6)	0.0269 (6)	0.0242 (6)	−0.0028 (5)	0.0017 (4)	−0.0025 (5)
C17	0.0272 (6)	0.0221 (6)	0.0213 (5)	0.0004 (5)	0.0080 (5)	0.0001 (4)
C18	0.0211 (5)	0.0264 (6)	0.0244 (6)	0.0012 (5)	0.0041 (4)	0.0016 (5)
C19	0.0233 (6)	0.0248 (6)	0.0216 (5)	−0.0018 (4)	0.0033 (4)	0.0001 (4)

### 4-[2-(4-Hydroxyphenyl)ethyl]-4-azatetracyclo[5.3.2.02,6.08,10]dodec-11-ene-3,5-dione (II) Geometric parameters (Å, º)

**Table:**

O1—C1	1.2208 (15)	C9—H9	1.0000
O2—C2	1.2115 (15)	C9—C10	1.5119 (17)
O3—C17	1.3713 (15)	C9—C11	1.5079 (18)
O3—H3	0.92 (2)	C10—H10	1.0000
N1—C1	1.3774 (15)	C10—C11	1.5047 (18)
N1—C2	1.3916 (15)	C11—H11A	0.9900
N1—C12	1.4607 (15)	C11—H11B	0.9900
C1—C3	1.5118 (16)	C12—H12A	0.9900
C2—C4	1.5083 (16)	C12—H12B	0.9900
C3—H3A	1.0000	C12—C13	1.5263 (17)
C3—C4	1.5432 (16)	C13—H13A	0.9900
C3—C8	1.5585 (17)	C13—H13B	0.9900
C4—H4	1.0000	C13—C14	1.5096 (16)
C4—C5	1.5572 (16)	C14—C15	1.3947 (17)
C5—H5	1.0000	C14—C19	1.3929 (17)
C5—C6	1.5041 (16)	C15—H15	0.9500
C5—C10	1.5336 (17)	C15—C16	1.3890 (17)
C6—H6	0.9500	C16—H16	0.9500
C6—C7	1.3275 (17)	C16—C17	1.3907 (17)
C7—H7	0.9500	C17—C18	1.3891 (17)
C7—C8	1.5042 (17)	C18—H18	0.9500
C8—H8	1.0000	C18—C19	1.3857 (17)
C8—C9	1.5333 (17)	C19—H19	0.9500
			
C17—O3—H3	107.2 (15)	C11—C9—H9	117.0
C1—N1—C2	112.97 (10)	C11—C9—C10	59.77 (8)
C1—N1—C12	124.18 (10)	C5—C10—H10	116.7
C2—N1—C12	122.81 (10)	C9—C10—C5	110.48 (10)
O1—C1—N1	123.86 (11)	C9—C10—H10	116.7
O1—C1—C3	127.16 (11)	C11—C10—C5	122.63 (10)
N1—C1—C3	108.98 (10)	C11—C10—C9	59.98 (8)
O2—C2—N1	123.37 (11)	C11—C10—H10	116.7
O2—C2—C4	127.95 (11)	C9—C11—H11A	117.7
N1—C2—C4	108.67 (10)	C9—C11—H11B	117.7
C1—C3—H3A	110.3	C10—C11—C9	60.25 (8)
C1—C3—C4	104.61 (9)	C10—C11—H11A	117.7
C1—C3—C8	111.96 (10)	C10—C11—H11B	117.7
C4—C3—H3A	110.3	H11A—C11—H11B	114.9
C4—C3—C8	109.35 (9)	N1—C12—H12A	109.1
C8—C3—H3A	110.3	N1—C12—H12B	109.1
C2—C4—C3	104.76 (9)	N1—C12—C13	112.65 (10)
C2—C4—H4	110.3	H12A—C12—H12B	107.8
C2—C4—C5	111.57 (10)	C13—C12—H12A	109.1
C3—C4—H4	110.3	C13—C12—H12B	109.1
C3—C4—C5	109.58 (9)	C12—C13—H13A	109.6
C5—C4—H4	110.3	C12—C13—H13B	109.6
C4—C5—H5	111.6	H13A—C13—H13B	108.2
C6—C5—C4	106.92 (9)	C14—C13—C12	110.09 (10)
C6—C5—H5	111.6	C14—C13—H13A	109.6
C6—C5—C10	110.41 (10)	C14—C13—H13B	109.6
C10—C5—C4	104.31 (9)	C15—C14—C13	122.11 (11)
C10—C5—H5	111.6	C19—C14—C13	119.93 (11)
C5—C6—H6	122.7	C19—C14—C15	117.88 (11)
C7—C6—C5	114.68 (11)	C14—C15—H15	119.4
C7—C6—H6	122.7	C16—C15—C14	121.23 (11)
C6—C7—H7	122.7	C16—C15—H15	119.4
C6—C7—C8	114.59 (11)	C15—C16—H16	120.1
C8—C7—H7	122.7	C15—C16—C17	119.86 (11)
C3—C8—H8	111.6	C17—C16—H16	120.1
C7—C8—C3	106.99 (10)	O3—C17—C16	118.25 (11)
C7—C8—H8	111.6	O3—C17—C18	122.08 (11)
C7—C8—C9	110.59 (10)	C18—C17—C16	119.66 (11)
C9—C8—C3	104.27 (10)	C17—C18—H18	120.1
C9—C8—H8	111.6	C19—C18—C17	119.81 (11)
C8—C9—H9	117.0	C19—C18—H18	120.1
C10—C9—C8	110.33 (10)	C14—C19—H19	119.2
C10—C9—H9	117.0	C18—C19—C14	121.51 (11)
C11—C9—C8	121.86 (11)	C18—C19—H19	119.2
			
O1—C1—C3—C4	−178.30 (11)	C5—C6—C7—C8	−0.72 (16)
O1—C1—C3—C8	−59.98 (15)	C5—C10—C11—C9	96.31 (12)
O2—C2—C4—C3	178.33 (12)	C6—C5—C10—C9	52.63 (13)
O2—C2—C4—C5	59.86 (16)	C6—C5—C10—C11	−14.10 (16)
O3—C17—C18—C19	177.36 (10)	C6—C7—C8—C3	−58.08 (14)
N1—C1—C3—C4	0.97 (12)	C6—C7—C8—C9	54.89 (14)
N1—C1—C3—C8	119.29 (10)	C7—C8—C9—C10	−51.60 (13)
N1—C2—C4—C3	−0.39 (12)	C7—C8—C9—C11	14.71 (16)
N1—C2—C4—C5	−118.87 (10)	C8—C3—C4—C2	−120.42 (10)
N1—C12—C13—C14	−176.50 (10)	C8—C3—C4—C5	−0.61 (12)
C1—N1—C2—O2	−177.71 (11)	C8—C9—C10—C5	−0.86 (14)
C1—N1—C2—C4	1.08 (13)	C8—C9—C10—C11	115.82 (12)
C1—N1—C12—C13	97.13 (13)	C8—C9—C11—C10	−96.40 (12)
C1—C3—C4—C2	−0.34 (12)	C10—C5—C6—C7	−54.04 (14)
C1—C3—C4—C5	119.47 (10)	C11—C9—C10—C5	−116.68 (11)
C1—C3—C8—C7	−59.82 (12)	C12—N1—C1—O1	0.18 (18)
C1—C3—C8—C9	−177.03 (9)	C12—N1—C1—C3	−179.12 (10)
C2—N1—C1—O1	177.99 (11)	C12—N1—C2—O2	0.13 (19)
C2—N1—C1—C3	−1.31 (13)	C12—N1—C2—C4	178.92 (10)
C2—N1—C12—C13	−80.46 (14)	C12—C13—C14—C15	−92.81 (13)
C2—C4—C5—C6	60.69 (12)	C12—C13—C14—C19	83.66 (13)
C2—C4—C5—C10	177.68 (9)	C13—C14—C15—C16	175.06 (11)
C3—C4—C5—C6	−54.85 (12)	C13—C14—C19—C18	−175.00 (11)
C3—C4—C5—C10	62.13 (11)	C14—C15—C16—C17	−0.43 (18)
C3—C8—C9—C10	63.09 (12)	C15—C14—C19—C18	1.62 (17)
C3—C8—C9—C11	129.40 (12)	C15—C16—C17—O3	−177.24 (10)
C4—C3—C8—C7	55.64 (12)	C15—C16—C17—C18	2.23 (18)
C4—C3—C8—C9	−61.57 (11)	C16—C17—C18—C19	−2.10 (18)
C4—C5—C6—C7	58.84 (14)	C17—C18—C19—C14	0.15 (18)
C4—C5—C10—C9	−61.91 (12)	C19—C14—C15—C16	−1.48 (18)
C4—C5—C10—C11	−128.65 (12)		

### 4-[2-(4-Hydroxyphenyl)ethyl]-4-azatetracyclo[5.3.2.02,6.08,10]dodec-11-ene-3,5-dione (II) Hydrogen-bond geometry (Å, º)

**Table:**

*D*—H···*A*	*D*—H	H···*A*	*D*···*A*	*D*—H···*A*
O3—H3···O1^i^	0.92 (2)	1.98 (2)	2.8955 (13)	172 (2)
C13—H13*A*···O2^ii^	0.99	2.52	3.4396 (16)	154
C6—H6···O1^iii^	0.95	2.50	3.4473 (15)	176

Symmetry codes: (i) −*x*, −*y*+1, −*z*+1; (ii) *x*−1, *y*, *z*; (iii) *x*+1/2, −*y*+1/2, *z*+1/2.
